# Effects of 17β-Estradiol Pollution on Microbial Communities and Methane Emissions in Aerobic Water Bodies

**DOI:** 10.3390/toxics12050373

**Published:** 2024-05-19

**Authors:** Zihao Gao, Yu Zheng, Zhendong Li, Aidong Ruan

**Affiliations:** 1 The National Key Laboratory of Water Disaster Prevention, Hohai University, Nanjing 210098, China; 211301020001@hhu.edu.cn (Z.G.); 211301010120@hhu.edu.cn (Y.Z.); 231301020003@hhu.edu.cn (Z.L.); 2 College of Hydrology and Water Resources, Hohai University, Nanjing 210098, China; 3 College of Geography and Remote Sensing, Hohai University, Nanjing 210098, China

**Keywords:** aquatic pollutant, bacterial and archaeal community, aerobic methane production, aquatic environment

## Abstract

17β-Estradiol (E2) is a widely present trace pollutant in aquatic environments. However, its impact on microbial communities in aerobic lake waters, which are crucial for methane (CH_4_) production, remains unclear. This study conducted an E2 contamination experiment by constructing laboratory-simulated aerobic microecosystems. Using 16S rRNA high-throughput sequencing, the effects of E2 on bacterial and archaeal communities were systematically examined. Combined with gas chromatography, the patterns and mechanisms of E2’s impact on CH_4_ emissions in aerobic aquatic systems were uncovered for the first time. Generally, E2 contamination increased the randomness of bacterial and archaeal community assemblies and weakened microbial interactions. Furthermore, changes occurred in the composition and ecological functions of bacterial and archaeal communities under E2 pollution. Specifically, two days after exposure to E2, the relative abundance of *Proteobacteria* in the low-concentration (L) and high-concentration (H) groups decreased by 6.99% and 4.01%, respectively, compared to the control group (C). Conversely, the relative abundance of *Planctomycetota* was 1.81% and 1.60% higher in the L and H groups, respectively. E2 contamination led to an increase in the relative abundance of the *methanogenesis* functional group and a decrease in that of the *methanotrophy* functional group. These changes led to an increase in CH_4_ emissions. This study comprehensively investigated the ecotoxicological effects of E2 pollution on microbial communities in aerobic water bodies and filled the knowledge gap regarding aerobic methane production under E2 contamination.

## 1. Introduction

According to reports, 17β-Estradiol (E2) is considered one of the most potent natural estrogens [1]. Its widespread presence in various aquatic environments has sparked global concerns as it can have significant impacts on aquatic organisms and human health, even at extremely low concentrations [2,3,4,5].

Current research has primarily focused on exploring the impacts of estrogens on higher organisms, while comparatively less attention has been given to investigating the ecotoxicological effects of estrogens on microorganisms. However, recent studies have confirmed that E2 has significant impacts on microbial communities in various environments. For instance, Chun et al. [6] conducted E2 contamination experiments in laboratory soil, revealing that E2 can alter the soil microbial community, with effects correlating with soil properties. Zhang et al. [7] suggested that E2 in soil may act as a nutrient for microbes, thereby stimulating the growth of certain bacteria. In river water, E2 concentrations ranging from 1 to 100 ng/L were found to significantly enhance the growth of heterotrophic nitrifying bacteria [8]. Additionally, the growth characteristics of *Enterobacteriaceae* were observed to change under E2 pollution [9]. However, information regarding the influence of E2 on microbial communities in aerobic lake waters remains extremely limited.

Water microbial communities play a crucial role in driving elemental biogeochemical cycles, facilitating the cycling of carbon, nitrogen, sulfur, phosphorus, and other essential elements [10,11]. Lakes, due to their capacity for storage, transport, and transformation of substantial carbon quantities [12], have emerged as focal points for carbon cycling dynamics and greenhouse gas emissions. Despite covering less than 4% of the Earth’s surface, lake ecosystems significantly contribute to methane (CH_4_) emissions, exerting pivotal influences on the global carbon cycle [13]. CH_4_ is not only a primary component of greenhouse gases but can also accumulate substantially in lakes, potentially leading to gas eruptions that cause significant human and animal fatalities. For instance, Lake Kivu in East Africa, known for its high CH_4_ content in deep, anoxic waters, is considered one of the most dangerous freshwater lakes [14]. Traditionally, CH_4_ production in lakes has been attributed primarily to methanogens in anaerobic environments. However, in recent years, evidence has been accumulating regarding CH_4_ production in aerobic water bodies [15,16], a phenomenon known as the “methane paradox”. Early studies on the “methane paradox” primarily focused on marine environments, proposing various hypotheses based on mechanisms involving methanogenic microorganisms to explain CH_4_ supersaturation in the presence of oxygen [17,18,19]. Recent research suggests that aerobic microbial conversion of methylphosphonate (MPn) may be a significant contributor to CH_4_ production in marine and freshwater environments [20,21,22], providing direct evidence for the existence of aerobic methanogenic microorganisms. Our previous studies showed that E2 significantly influenced CH_4_ emission rates in both simulated natural and anaerobic systems, with its effects being constrained by major environmental factors [23,24,25]. However, the influence of E2 on CH_4_ emissions in aerobic lake waters remains unclear.

Based on these considerations, this study established aerobic simulated microecosystems in the laboratory. Different concentrations of E2 were added to the systems to conduct pollution experiments aiming to: (1) investigate the impact of E2 pollution on CH_4_ emissions in aerobic lake waters; (2) assess the ecotoxicological effects of E2 pollution on microbial communities in aerobic lake waters; and (3) elucidate the microbiological mechanisms by which E2 affects CH_4_ emissions. These findings will provide a theoretical basis for future water pollution control and aid in more accurately predicting and assessing methane emissions in lake water bodies.

## 2. Materials and Methods

### 2.1. Experimental Design

The sediment and overlying water samples used in this study were obtained from Longxu Lake in Anhui Province, China, an ecologically protected area where no estrogen was detected. The sediment samples underwent a series of treatments including air-drying, grinding, sieving (100 mesh), and homogenization. Methylphosphonic acid was added to the water samples to achieve a final concentration of 1 mmol/L. Approximately 100 g of treated sediment and 150 mL of treated overlying water were placed in 500 mL conical flasks. Subsequently, these flasks were covered with membranes (air flux: 1020 m^3^/m^2^·h at 0.01 MPa) with a pore size of 0.2–0.3 μm, ensuring system aeration while preventing the introduction of external microbes. Nine laboratory-simulated aerobic microecosystems were established using the described method and were placed in constant temperature incubators at 30 °C, shielded from light, well-ventilated, and agitated at 100 rpm. After three days, the gas emission rates of each system stabilized and exhibited uniformity; this was followed by the initiation of E2 pollution treatment on the systems. Stock solutions of E2 were prepared by dissolving E2 (99%, CAS 50-28-2, Thermo Scientific, Waltham, MA, USA) in ethanol. Volumes of 30 µL of these stock solutions were added at different concentrations to the systems to achieve final E2 concentrations of 0 ng/L (control group, C), 100 ng/L (low-concentration group, L), and 10,000 ng/L (high-concentration group, H). Each group consisted of three replicate samples.

### 2.2. Sample Collection and Measurement

The date of E2 solution addition was designated as Day 0. Prior to E2 contamination, gas and slurry–water mixture samples were collected once. Subsequently, gas samples were collected every 24 h, and slurry–water mixture samples were collected every 48 h. The specific collection method involved using a sterile syringe to puncture the septum and collect 5 mL of headspace gas samples, followed by sealing the system for incubation. After 2.5 h of incubation, another 5 mL of headspace gas samples were collected using a sterile syringe. Then, each system was thoroughly mixed, and 10 mL of slurry–water mixture samples were collected and stored in a −80 °C freezer.

The experimental period lasted for 7 days. Gas samples collected daily were analyzed for CH_4_ concentration using gas chromatography (GC-7890B, Agilent Technologies, Santa Clara, CA, USA). A total of 36 slurry–water mixture samples collected on the 0th, 2nd, 4th, and 6th days were subjected to high-throughput sequencing.

### 2.3. DNA Extraction, Amplification, and Sequencing

The DNA from the slurry–water mixture samples was extracted using the TGuide S96 Magnetic Soil/Stool DNA Kit (Tiangen Biotech (Beijing) Co., Ltd., Beijing, China). Subsequently, the DNA concentration of the samples was measured using the Qubit dsDNA HS Assay Kit and Qubit 4.0 Fluorometer (Invitrogen, Thermo Fisher Scientific, Waltham, MA, USA). The V4-V5 region of the 16S rRNA gene was amplified in each sample using the 515F primer (5′-GTGYCAGCMGCCGCGGTAA-3′) and the 926R primer (5′-CCGYCAATTYMTTTRAGTTT-3′). Sequencing adapters were attached to the ends of the primers for PCR amplification, and the resulting products underwent purification, quantification, and normalization to create sequencing libraries. After passing quality control assessment, the libraries were sequenced using Illumina Novaseq 6000 (Illumina, Santiago, CA, USA). Additionally, raw data have been uploaded to the NCBI SRA database (No. PRJNA1097048).

### 2.4. Bioinformatic Analysis

The raw reads obtained from sequencing were filtered using Trimmomatic (version 0.33). Primer sequences were identified and removed to obtain clean reads using Cutadapt (version 1.9.1). Subsequently, the clean reads underwent feature classification using dada2, resulting in the generation of amplicon sequence variants (ASVs) [26]. ASVs with relative abundances of less than 0.005% were filtered out. Taxonomic annotation of the filtered ASVs was conducted using the Naive Bayes classifier [27] based on the Silva.138 reference database [28].

Alpha diversity indices of the samples were calculated using the QIIME2 software [27]. Beta diversity was evaluated through Principal Coordinates Analysis (PCoA) based on the Bray–Curtis distance [29]. The relative importance of microbial community assembly processes was determined using the iCAMP model [30]. Molecular Ecological Networks (MENs) were established using Random Matrix Theory (RMT), and subsequent analysis was carried out with the Molecular Ecological Network Analysis Pipeline (MENAP, http://mem.rcees.ac.cn:8081 accessed on 1 May 2023) [31]. Gephi (version 0.9.2) was utilized for visualizing all networks. Functional groups within the samples were predicted using FAPROTAX [32], and the results were visualized with the R package pheatmap to generate heat maps. Redundancy analysis (RDA) was conducted using vegan (version 2.3-0), with significance tested via Monte Carlo permutation tests (permu = 999).

### 2.5. Analysis of Methane Emission Rates

The specific formula for calculating CH_4_ emission rates is as follows:

v = ((c_2_ − c_1_) × V_h_ × 1/22.4 × 273/(273 + T) × P/101325)/(V_s_ × t), (1)

v: the CH_4_ emission rate (μmol·L^−1^·h^−1^);

c_1_: the CH_4_ volume concentration before sealing (ppm);

c_2_: the CH_4_ volume concentration after 2.5 h of sealing (ppm);

V_h_: the headspace volume (mL);

T: the gas temperature (°C);

P: the gas pressure (Pa);

V_s_: the sample volume (mL);

t: the sealing time (h).

### 2.6. Statistical Analysis

Permutational Multivariate Analysis of Variance (PERMANOVA) was utilized to examine disparities in microbial community structures among different groups. The Student’s *t*-test was employed to assess the statistical significance of differences between two samples. Differences were considered statistically significant if the *p*-value was less than 0.05.

## 3. Results

### 3.1. The Impact of 17β-Estradiol Pollution on Methane Emission Patterns

**Figure 1:**
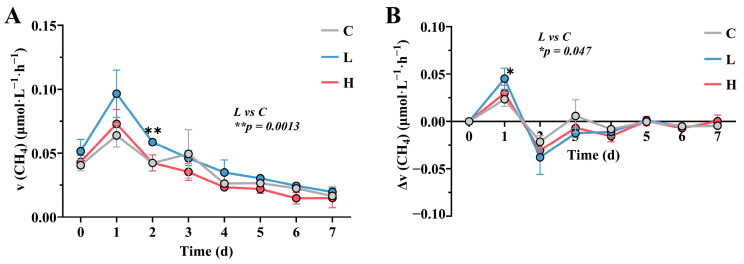
Temporal changes in methane emission rate (**A**) and rate increment (**B**). The rate increment is calculated by subtracting the previous day’s rate from the current day’s rate. C represents the control group, L represents the low-concentration group, and H represents the high-concentration group. The significance markers in Figure 1A and 1B represent the results of the Student’s *t*-test comparing the C and L groups on day 2 and day 1, respectively.

The study tracked the changes in CH_4_ emission rates within each treatment group over 7 days post E2 pollution (Figure 1A). The results revealed differences in CH_4_ emission rates among the groups only during the first 2 days, with the low-concentration group significantly higher than the control group on day 2 (*p* = 0.0013). Subsequently, from days 3 to 7, all groups showed a gradual decline in CH_4_ emissions without significant discrepancies. To further investigate the inter-group disparities, an analysis of CH_4_ emission rate increments was performed (Figure 1B). Within the first 2 days, fluctuations were observed in the rate increments across all treatment groups. Notably, on day 1, both pollution groups had higher rate increments compared to the control group, with particularly the low-concentration group displaying a significant increase over the control group (*p* = 0.047). By days 3 to 7, CH_4_ emissions had stabilized in all groups, with rate increments approaching zero. In conclusion, E2 was found to stimulate short-term CH_4_ production in aerobic water systems.

### 3.2. Response of Bacterial and Archaeal Community Diversity to 17β-Estradiol Pollution

**Figure 2:**
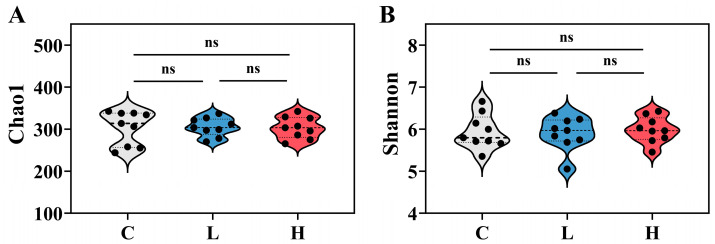
Chao1 (**A**) and Shannon (**B**) indices of bacterial and archaeal communities in each group after E2 contamination. C represents the total of samples from the control group collected on days 2, 4, and 6; L represents the total of samples from the low-concentration group collected on days 2, 4, and 6; H represents the total of samples from the high-concentration group collected on days 2, 4, and 6. “ns” indicates no significant difference.

Sequencing of 36 samples yielded a total of 562 ASVs, classified into 25 phyla, 133 families, and 186 genera. Bacteria accounted for 82.55–99.54%, archaea for 0.04–8.94%, and unassigned organisms for 0.42–17.29% of the community. The study treated bacterial and archaeal communities as a unified entity. Following E2 pollution, there were no significant differences in Chao1 and Shannon indices of bacterial and archaeal communities among the three treatment groups (Figure 2A,B). This indicates that E2 pollution did not significantly impact the species richness and diversity of bacterial and archaeal communities within the system.

**Figure 3:**
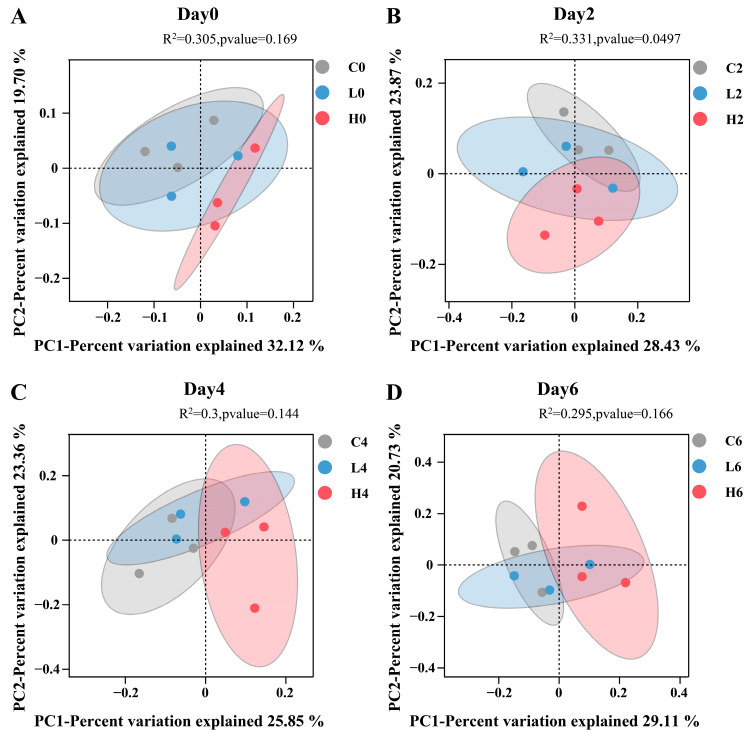
The Principal Coordinate Analysis (PCoA) of bacterial and archaeal communities on days 0 (**A**), 2 (**B**), 4 (**C**), and 6 (**D**). Sample labeling rule: Letters represent groups, and numbers represent dates. For example, C0 represents samples from the control group on day 0.

To evaluate the impact of E2 on bacterial and archaeal community structure, we conducted Principal Coordinate Analysis (PCoA) on the community compositions of the three treatment groups on different dates. The results showed no significant differences in community structures among the three treatment groups on day 0 (Figure 3A), indicating homogeneity before E2 pollution. However, on day 2, a significant difference emerged between the control group and the two pollution groups (PERMANOVA, *p* = 0.0497) (Figure 3B), suggesting a notable effect of E2. On days 4 and 6, the community structures of all three groups were similar, showing no significant differences (Figure 3C,D). Overall, these findings suggest that under aerobic conditions, the influence of E2 on bacterial and archaeal communities may be transient.

### 3.3. Exposure to 17β-Estradiol Alters Taxonomic Composition of Bacterial and Archaeal Communities

**Figure 4:**
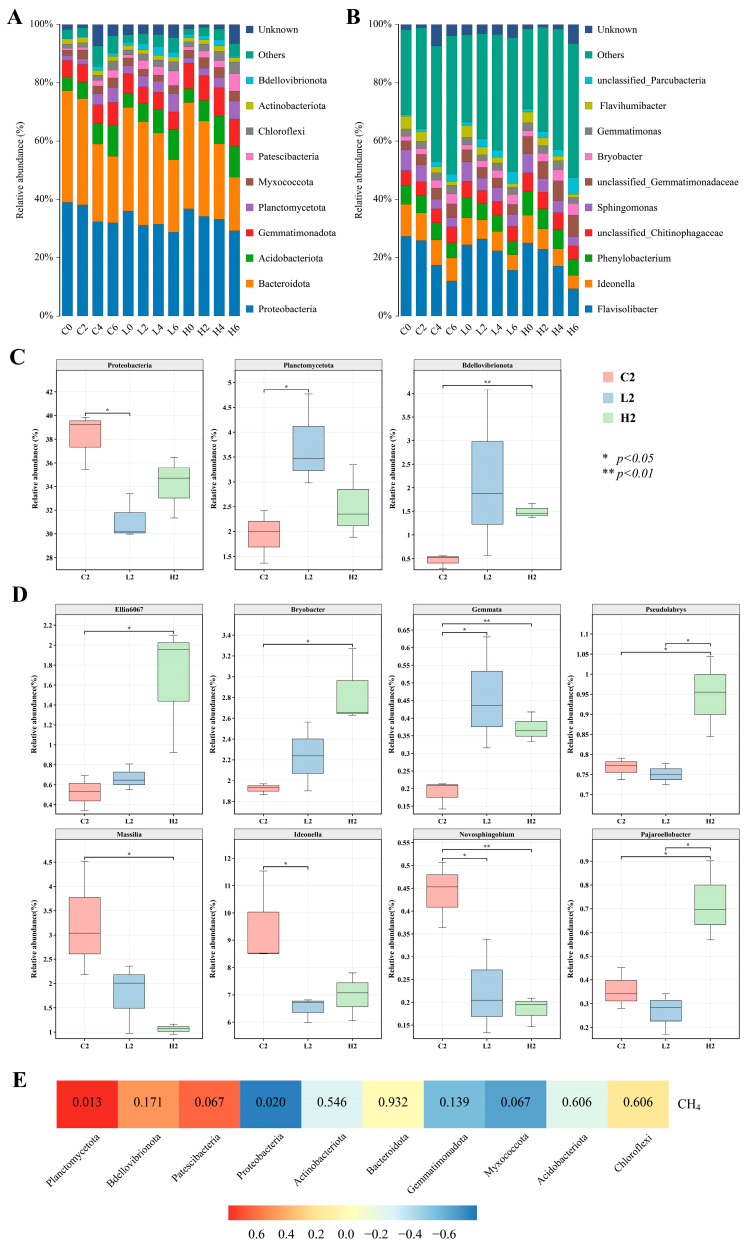
Taxonomic composition of bacterial and archaeal communities at the phylum (**A**) and genus (**B**) levels, and relative abundance of differential phyla (**C**) and genera (**D**) on day 2. C2, L2, and H2 represent samples from the control group, low-concentration group, and high-concentration group, respectively, on day 2. (**E**) Heatmap of the correlation between major phyla and CH_4_ emission rates on day 2.

Exposure to E2 significantly impacted the composition of bacterial and archaeal communities. During E2 pollution, *Proteobacteria* (29.3–39.0%) and *Bacteroidota* (18.3–38.3%) were dominant in all three treatment groups (Figure 4A). However, the relative abundance of these dominant phyla gradually decreased over the incubation period. To compare the differences in community compositions among the three groups, particular attention was given to analyzing the second day, where significant differences in community structure were observed. Analysis of variance (ANOVA) revealed that on day 2, three phyla among the top ten in relative abundance showed significant differences among the groups. In the two pollution groups, the relative abundance of *Proteobacteria* was lower than that of the control group, especially with the low-concentration group showing a significant decrease compared to the control group (Figure 4C). This suggests that the addition of E2 reduced the dominance of *Proteobacteria*. Furthermore, in the pollution groups, the relative abundances of *Planctomycetota* and *Bdellovibrionota* were higher than those in the control group, especially with *Planctomycetota* in the low-concentration group and *Bdellovibrionota* in the high-concentration group showing significantly higher relative abundances than in the control group. Therefore, E2 significantly increased the relative abundances of *Planctomycetota* and *Bdellovibrionota* in aerobic water bodies.

At the genus level, *Flavisolibacter* was consistently the most abundant genus in both the control group and low-concentration group throughout the experiment (12.1–27.3%, 15.8–26.5%), followed by *Ideonella* (7.9–11.0%, 5.2–9.2%) (Figure 4B). In the high-concentration group, *Flavisolibacter* remained dominant in relative abundance (9.4–25.1%), but *Ideonella* dropped to fourth and seventh place on day 4 and day 6, indicating a threat to the dominance of *Ideonella* posed by high concentrations of E2. Particularly noteworthy is that on day 2, *Ideonella* in the low-concentration group was significantly lower than in the control group (Figure 4D), suggesting that even low concentrations of E2 reduced the dominance of *Ideonella*. Additionally, in both pollution groups, *Ellin6067*, *Bryobacter*, and *Gemmata* had higher relative abundances compared to the control group, with this difference being more pronounced in the high-concentration group (Figure 4D). Conversely, *Massilia* and *Novosphingobium* had lower relative abundances in both pollution groups, with a more significant decrease observed in the high-concentration group (Figure 4D). This indicates that E2 significantly increased the relative abundances of *Ellin6067*, *Bryobacter*, and *Gemmata* while markedly decreasing those of *Massilia* and *Novosphingobium*, particularly under high-concentration conditions. Furthermore, *Pseudolabrys* and *Pajaroellobacter* showed no significant differences in relative abundance compared to the control group in the low-concentration group but exhibited significantly higher relative abundances in the high-concentration group (Figure 4D), indicating that E2 only promotes the growth of *Pseudolabrys* and *Pajaroellobacter* at high concentrations.

### 3.4. The Influence of 17β-Estradiol Pollution on Community Assembly

**Figure 5:**
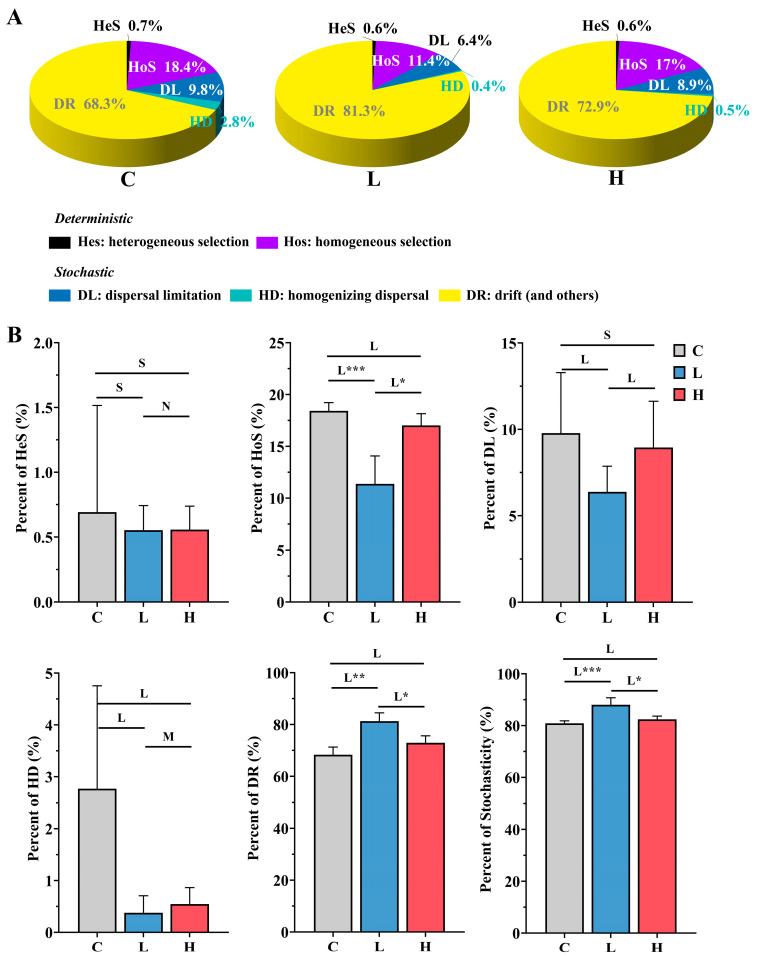
Relative importance of various ecological processes within each group (**A**) and differences in ecological processes among groups (**B**) after E2 pollution. C represents the total of samples from the control group collected on days 2, 4, and 6; L represents the total of samples from the low-concentration group collected on days 2, 4, and 6; H represents the total of samples from the high-concentration group collected on days 2, 4, and 6. *** *p* < 0.001; ** *p* < 0.01; * *p* < 0.05. L, M, S, and N represent large (|d| > 0.8), medium (0.5 < |d| ≤ 0.8), small (0.2 < |d| ≤ 0.5), and negligible (|d| ≤ 0.2) effect sizes of E2 pollution based on Cohen’s d.

iCAMP was used to quantify the assembly of bacterial and archaeal communities after adding E2 solution. The dominant process across all three treatment groups was drift (DR, 68.3–81.3%) in stochastic processes, followed by homogeneous selection (HoS, 11.4–18.4%) in deterministic processes (Figure 5A). Consequently, the assembly of bacterial and archaeal communities in all groups was primarily governed by stochastic processes. Nevertheless, variations were observed among the ecological processes within the three treatment groups. Specifically, in the low-concentration group, HoS was significantly lower compared to the control group (Cohen’s d = 3.53, *p* = 0.0004), while DR was significantly higher (Cohen’s d = −4.27, *p* = 0.002) (Figure 5B). Similarly, in the high-concentration group, HoS and DR displayed comparable trends to the control group, although the differences were not statistically significant. These findings suggest that E2 significantly impacts the principal ecological processes of bacterial and archaeal communities within the system, leading to a notable increase in the stochasticity of community assembly (Cohen’s d = −3.57, *p* = 0.0004) (Figure 5B).

### 3.5. Molecular Ecological Network Analysis

**Figure 6:**
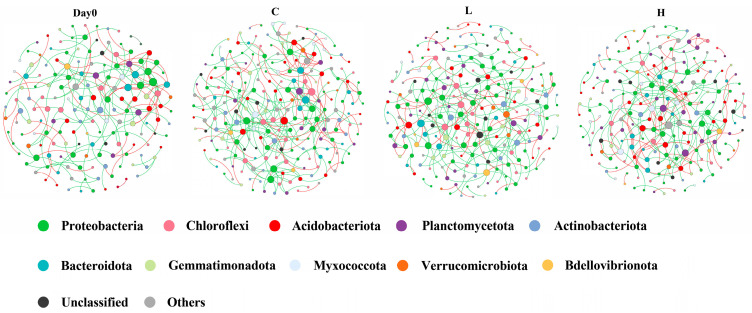
Molecular ecological networks (MENs) before and after E2 pollution. Node size represents the node degree. Edge color represents positive (red) and negative (green) correlations. Day 0 represents the total of samples from three groups collected on day 0; C represents the total of samples from the control group collected on days 2, 4, and 6; L represents the total of samples from the low-concentration group collected on days 2, 4, and 6; H represents the total of samples from the high-concentration group collected on days 2, 4, and 6.

The impact of E2 on microbial interactions within bacterial and archaeal communities was revealed through molecular ecological network analysis (MENs). The visualization of networks before and after E2 pollution is shown in Figure 6, with specific network properties detailed in Table S1. The node connectivity of the four networks conformed to a power-law distribution (R^2^ = 0.89–0.95), indicating that these networks were all scale-free networks. Additionally, with average path lengths ranging from 6.39 to 6.92 and close approximation of the logarithm of the total number of nodes, the networks exhibited typical small-world characteristics. Furthermore, all the networks demonstrated modularity values between 0.774 and 0.804, significantly higher than those of corresponding random networks, indicating the presence of modular features in the constructed networks.

Compared to the initial state, the three treatment groups exhibited a significant increase in network nodes and links following the addition of E2 solution, indicating an enhancement in network complexity. This improvement may be attributed to ethanol acting as a solvent, resulting in more nutrients being provided to bacteria and archaea, thereby boosting microbial activity and diversity and, consequently, enhancing network complexity. In contrast to the control group, the two pollution groups showed notably fewer links, suggesting simpler network structures. Furthermore, both pollution groups had lower average degree (avgK) and average clustering coefficient (avgCC) values than the control group, indicating weaker node connectivity and reduced closeness and clustering among nodes in pollution networks. These results suggest that E2 could reduce the complexity and stability of bacterial and archaeal community networks in aerobic water bodies.

Based on the within-module connectivity (Zi) and among-module connectivity (Pi), nodes are categorized into network hubs, module hubs, connectors, and peripherals [33]. The first three types are regarded as keystone taxa, playing a pivotal role in the system’s resilience against external disturbances or species invasions [34]. Each network has distinct connectors and module hubs (Figure S1). Before pollution, the network had three connectors. Following E2 contamination, the control group showed four module hubs and three connectors, the low-concentration group had four module hubs and eight connectors, and the high-concentration group had four module hubs and four connectors. Therefore, the introduction of E2 led to an increase in the number of keystone taxa within the network. The specific classification of these keystone ASVs is detailed in Table S2. In the control group, over half of the keystone ASVs belonged to *Proteobacteria*, indicating their potential importance as the predominant phylum in the system. However, in the low-concentration group, only two keystone ASVs were from *Proteobacteria*, accounting for 16.7% of the total keystone ASVs, while two keystone ASVs were identified as *Planctomycetota*, which were absent among the keystone taxa in the control group. In the high-concentration group, 50% of the keystone ASVs belonged to *Planctomycetota*. This suggests that with increasing E2 concentration, the interactions between *Proteobacteria* and other microorganisms gradually weaken, while the significance of *Planctomycetota* in the network increases. At the family level, both pollution groups had keystone ASVs belonging to *Gemmatimonadaceae*, *Gemmataceae*, and *Isosphaeraceae*. However, in the control group, there were no keystone ASVs from these three families. Therefore, the addition of E2 could enhance the interactions between *Gemmatimonadaceae*, *Gemmataceae*, *Isosphaeraceae*, and other microorganisms.

### 3.6. The Influence of 17β-Estradiol Contamination on Ecological Functions

**Figure 7:**
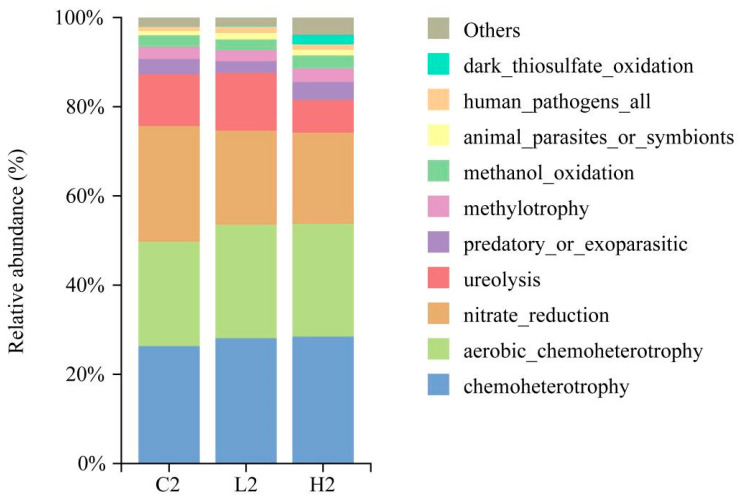
Relative abundance of functional groups in different treatment groups on day 2.

Functional predictions were conducted using Faprotax for bacterial and archaeal communities on day 2. Out of 562 ASVs, 83 were assigned to at least one functional group. The most abundant functional groups in all three treatment groups were *chemoheterotrophy* (26.3–28.5%), followed by *aerobic_chemoheterotrophy* (23.4–25.5%) and *nitrate_reduction* (20.4–25.9%) (Figure 7). Significant variations in the relative abundances of major functional groups were observed among the three treatment groups. Both pollution groups exhibited higher levels of *aerobic_chemoheterotrophy* and *chemoheterotrophy* compared to the control group, while *nitrate_reduction* was lower in the pollution groups (Figure S2). Moreover, functional groups associated with methane production, such as *methanogenesis*, *methanogenesis_by_CO_2__reduction_with_H_2_*, and *hydrogenotrophic_methanogenesis*, were most abundant in the high-concentration group, followed by the low-concentration group. The methane oxidation functional group, *methanotrophy*, was enriched in the control group (Figure S2). Hence, E2 has the potential to influence microbial carbon and nitrogen cycling within the system.

## 4. Discussion

### 4.1. Ecological Toxicological Effects of 17β-Estradiol on Bacterial and Archaeal Communities

Based on the findings, E2 has disrupted the original structure of bacterial and archaeal communities in aerobic water bodies. The molecular ecological network analysis revealed that both pollution groups exhibited lower links, avgK, and avgCC compared to the control group. This indicates that the introduction of E2 weakened interactions among microorganisms, impacting information flow and material cycling within the ecosystem, and consequently, reducing ecosystem stability. Additionally, the iCAMP analysis demonstrated a significant increase in the proportion of stochasticity in community assembly due to E2 pollution, suggesting heightened uncertainty in the formation and evolution of microbial community structures. This could be attributed to variations in species’ adaptability to E2. The introduction of E2 decreased the dominance of the major phylum *Proteobacteria*, providing additional resources and available space for other microorganisms, and thereby amplifying the randomness in community assembly [35]. Overall, within aerobic water bodies, E2 contamination led to microbial communities becoming more unpredictable and unstable.

However, the impact is only effective in the short term. Beta diversity analysis revealed significant differences in the bacterial and archaeal community structures of the three treatment groups only on the second day after E2 contamination, with no notable variances on the fourth and sixth days. This suggests that the influence of E2 contamination on the structure of bacterial and archaeal communities is transient. Over time, the community structure may gradually revert to its original state. This recovery capability could be attributed to keystone taxa within the community [31]. The number of keystone taxa in the pollution groups was notably higher than that in the control group, indicating that under the pressure of E2 contamination, specific microbial taxa began to assume more critical roles, exerting essential regulatory effects on maintaining the structure and function of the entire community [36]. Notably, the significance of *Planctomycetota* in the network increased gradually, with two family-level members, *Gemmataceae* and *Isosphaeraceae*, identified as specific keystone taxa in the pollution groups. These bacterial families exhibit robust hydrolytic potential, enabling them to utilize a broad spectrum of organic substances [37,38] and potentially participate in E2 degradation. Therefore, *Planctomycetota* may exhibit higher adaptability, enabling it to play a dominant role under E2 contamination and facilitate the community’s recovery towards a relatively stable state by degrading E2.

Moreover, E2 contamination altered the composition of bacterial and archaeal communities. Specifically, E2 significantly increased the relative abundance of *Ellin6067* and *Bryobacter*, with a more pronounced effect observed at higher concentrations. Additionally, high concentrations of E2 notably stimulated the growth of *Pseudolabrys*. *Bryobacter* exhibits chemoheterotrophic activity, enabling it to degrade organic compounds [39,40]. Research by Liu et al. has indicated that *Ellin6067* is capable of degrading organic pollutants [41]. Additionally, *Pseudolabrys* demonstrates strong adaptability to extreme environments [42] and possesses significant potential in removing nitrogen compounds, and degrading organic pollutants like chlorinated alkanes, chlorinated alkenes and benzoic acid [43]. These findings suggest that these three genera may participate in aerobic pathways for E2 degradation in aquatic environments. Overall, following E2 contamination, the relative abundance of bacteria associated with E2 degradation significantly increased, with a more pronounced effect observed at higher E2 concentrations. This indicates that E2 may be degraded by microorganisms as an organic pollutant in the system, thereby impacting bacterial and archaeal community structures.

### 4.2. Mechanism of 17β-Estradiol in Promoting Methane Emission

Traditionally, CH_4_ production was attributed primarily to anaerobic methanogenic archaea. However, evidence accumulated over the past three decades suggests that CH_4_ can also be generated in aerobic environments [15,16], a phenomenon termed the “methane paradox”. The “methane paradox” has been extensively documented and is considered to contribute significantly to the biogeochemical cycle of CH_4_ [44]. Currently, two main perspectives prevail regarding CH_4_ production under aerobic conditions: (1) Methanogens can survive in aerobic conditions by utilizing their self-synthesized antioxidant pathways, leading to the production of CH_4_ [45,46]. (2) Certain bacteria and fungi can metabolize methylphosphonic acid under aerobic conditions, producing CH_4_ through demethylation processes [20,21,22].

In the aerobic microecosystems, both the CH_4_ emission rate and the structure of the bacterial and archaeal communities were significantly affected within two days after E2 pollution. Therefore, CH_4_ emissions may be influenced by changes in the composition of bacterial and archaeal communities. This hypothesis was validated through Redundancy analysis (RDA), as depicted in Figure S3. The Monte Carlo test results indicate that both the CH_4_ emission rate and E2 concentration were significantly correlated with the structure of bacterial and archaeal communities (*p* < 0.05) (Table S3). This suggests that E2 has a noteworthy impact on the bacterial and archaeal communities, thereby affecting CH_4_ production.

The functional prediction results suggest that E2 increased the relative abundance of the *methanogenesis* functional group. The ASVs assigned to *methanogenesis* belonged to the genus *Methanoregula*. *Methanoregula* are hydrogenotrophic methanogens whose growth is inhibited even under low oxygen levels [47]. Additionally, studies have indicated that small molecular heat shock proteins play a crucial role in tolerating oxidative stress. The absence of these genes resulted in lower oxygen tolerance in *Methanoregula* [48]. Therefore, the presence of *Methanoregula* in the system could be attributed to anaerobic microenvironments created by sediment facilitating their survival. The higher relative abundance of the *methanogenesis* functional group in the pollution groups might suggest that E2 or its metabolites promoted the growth of *Methanoregula*, thus enhancing CH_4_ production.

To investigate the correlation between bacteria and CH_4_ production in aerobic systems, we utilized a heatmap to visualize the relationship between the relative abundance of the top ten phyla and CH_4_ emission rates (Figure 4E). The results demonstrated a significant positive correlation between *Planctomycetota* and CH_4_ emission rates. Methylphosphonic acid could be utilized by microorganisms through various pathways, but only the C-P cleavage pathway could release CH_4_ [49]. In *Escherichia coli*, the C-P cleavage pathway was encoded by 14 genes *(phnC-phnP*) [50,51]. Zhi et al. [52] found that *Planctomycetota* carried key genes involved in organic phosphonate metabolism, such as *phnM* and *phnI*. Therefore, *Planctomycetota* might have the potential to produce CH_4_ from methylphosphonic acid under aerobic conditions. The promoting effect of E2 pollution on the growth of *Planctomycetota* contributed to increased CH_4_ production in the system.

The emission of CH_4_ is the result of the combined processes of CH_4_ production and CH_4_ oxidation. The *methanotrophy* functional group, which is capable of consuming CH_4_, was enriched in the control group, indicating that the addition of E2 reduced the relative abundance of methanotrophic bacteria in the system, thereby decreasing CH_4_ consumption. The ASVs assigned to *methanotrophy* all belonged to *Proteobacteria*, and *Proteobacteria* exhibited a significant negative correlation with CH_4_ emission rates (Figure 4E). Additionally, E2 significantly decreased the dominance of *Proteobacteria*. Hence, the inhibitory effect of E2 on methanotrophic bacteria is also a key factor contributing to the higher CH_4_ emissions in the pollution groups.

## 5. Conclusions

In conclusion, E2 contamination significantly disrupted the community structure of bacteria and archaea in aerobic water bodies, leading to a reduction in microbial interactions and a notable increase in the stochasticity of community assembly. This resulted in heightened unpredictability and instability within the communities. Specifically, the most dominant *Proteobacteria* phylum experienced a decline in its advantageous position due to E2 pollution. Conversely, *Planctomycetota* demonstrated a strong adaptability to E2 contamination, as evidenced by a marked increase in relative abundance, and played a crucial role in community recovery. At the genus level, there was a substantial rise in the relative abundance of bacteria associated with E2 degradation, including *Ellin6067*, *Bryobacter*, and *Pseudolabrys*. Furthermore, E2 contamination promoted CH_4_ emissions through three pathways: stimulating the growth of *Methanoregula* in anaerobic microenvironments; boosting the abundance of *Planctomycetota* capable of utilizing methylphosphonate for methane production; and inhibiting the growth of methanotrophic bacteria. This study filled the theoretical gap between E2 metabolism and methane metabolism in aerobic waters and contributed to enriching the ecotoxicological theory of E2.

## Data Availability

Raw data have been uploaded to the NCBI SRA database (No. PRJNA1097048).

## Supplementary Materials

The following supporting information can be downloaded at: https://www.mdpi.com/article/10.3390/toxics12050373/s1. Figure S1: Zi-Pi plots for each network; Figure S2: Functional group abundance heatmap of bacterial and archaeal communities in different treatment groups on day 2; Figure S3: Redundancy analysis (RDA) of the correlation between ASVs of bacterial and archaeal communities and properties on day 2; Table S1: Topological properties of empirical networks and random networks; Table S2: Taxonomic classification of keystone ASVs; Table S3: Results of Monte Carlo permutation test.
