# Supplementary material for: Effects of 17β-Estradiol Pollution on Microbial Communities and Methane Emissions in Aerobic Water Bodies
Source: Toxics. 2024 May 19;12(5):373. doi: 10.3390/toxics12050373 (PMC11126138; doi:10.3390/toxics12050373)
Supplement: Supplementary file 1 [file toxics-12-00373-s001.zip › toxics-2984205-supplementary.pdf]

Effects of 17β-Estradiol Pollution on Microbial Communities and Methane Emissions in Aerobic Water Bodies

Zihao Gao [a, c], Yu Zheng [a, c], Zhendong Li[a, c], Aidong Ruan [a, b,*]

[a] The National Key Laboratory of Water Disaster Prevention, Hohai University, Nanjing 210098, China.

[b] College of Geography and Remote Sensing, Hohai University, Nanjing 210098, China.

[c] College of Hydrology and Water Resources, Hohai University, Nanjing 210098, China.

[*]Corresponding Author: Aidong Ruan

E-mail address: adruan@hhu.edu.cn

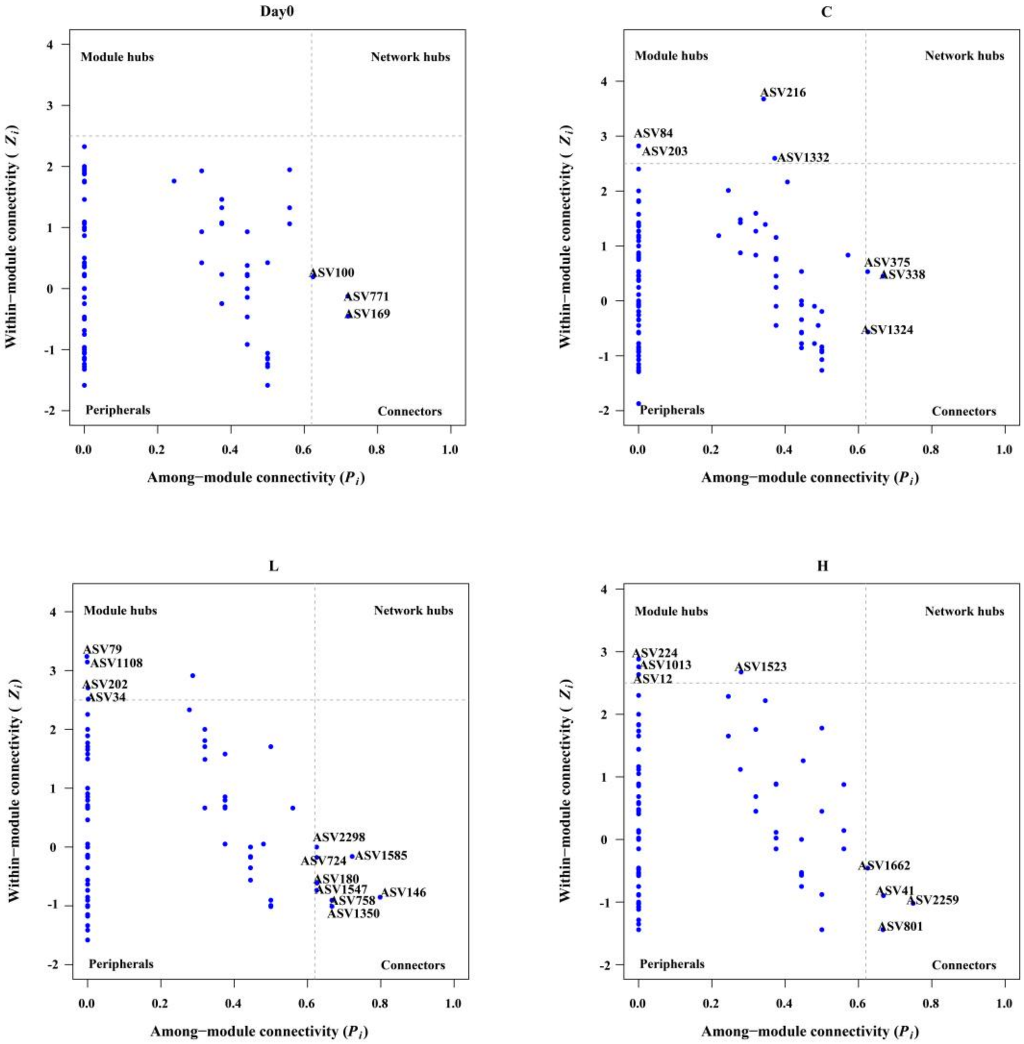

Figure S1 Zi-Pi plots for each network.

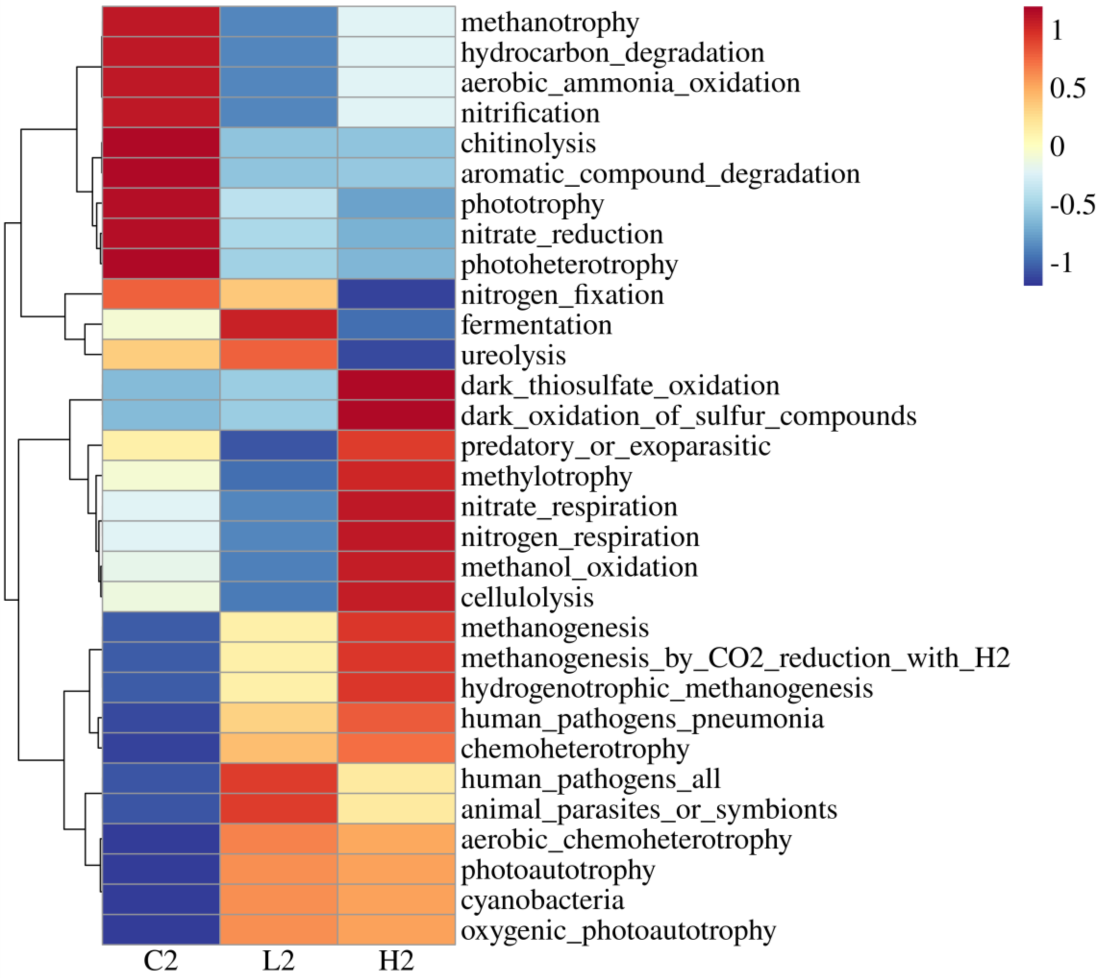

Figure S2 Functional group abundance heatmap of bacterial and archaeal communities in different treatment groups on day 2.

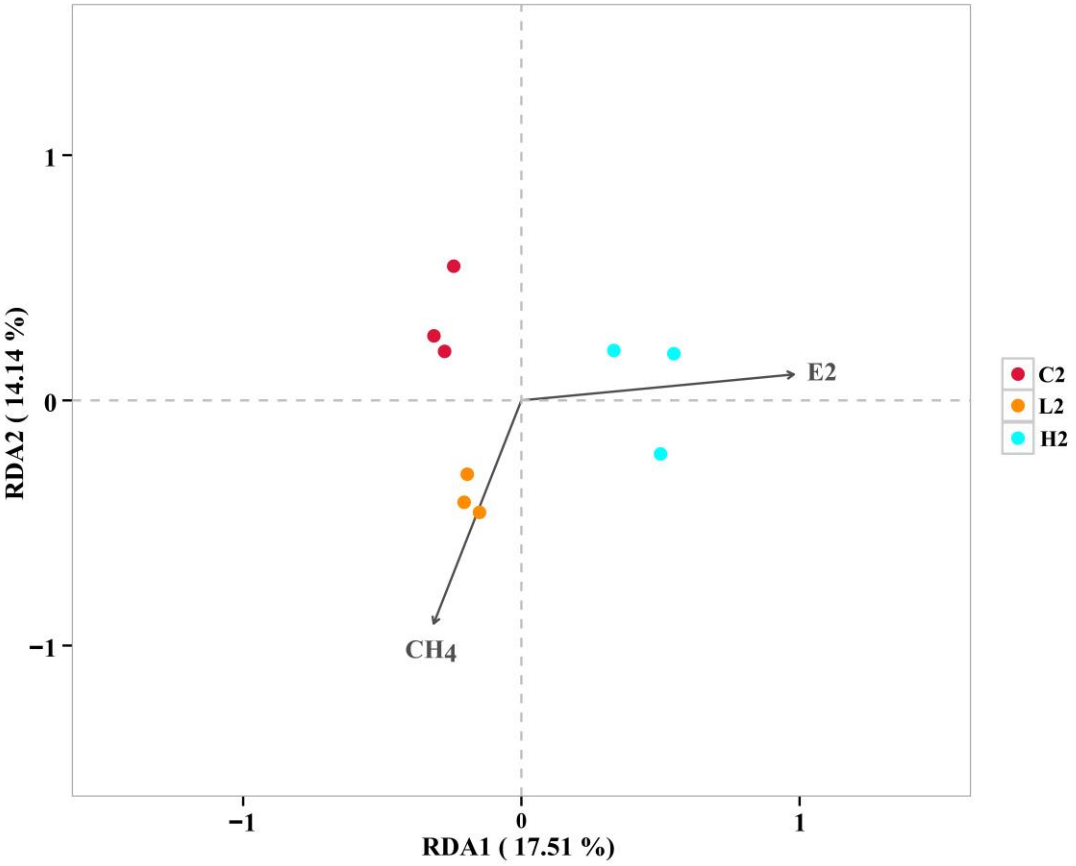

Figure S3 Redundancy analysis (RDA) of the correlation between ASVs of bacterial and archaeal communities and properties on day 2.

Table S1 Topological properties of empirical networks and random networks.

Group	Empirical networks									Random networks			
	**Cutoff**	**Total nodes**	**Total links**	**R square of power-law**	**Average degree (avgK)**	**Average clustering coefficient (avgCC)**	**Average path distance (GD)**	**Harmonic geodesic distance (HD)**	**Modularity (module numbers)**	**Average clustering coefficient (avgCC)**	**Average path distance (GD)**	**Harmonic geodesic distance (HD)**	**Modularity (M)**
Day0	0.92	165	190	0.94	2.30	0.06a	6.92b	5.12	0.774(25)c	0.002 ± 0.005	5.881 ± 0.245	4.797 ± 0.156	0.724 ± 0.011
C	0.92	222	324	0.91	2.92	0.16a	6.70b	5.28	0.781(23)c	0.004 ± 0.004	4.745 ± 0.094	4.121 ± 0.067	0.618 ± 0.009
L	0.92	225	266	0.89	2.36	0.10a	6.70b	5.41	0.802(31)c	0.001 ± 0.003	6.243 ± 0.218	5.199 ± 0.139	0.726 ± 0.009
H	0.92	209	267	0.95	2.56	0.14a	6.39b	5.00	0.804(29)c	0.002 ± 0.003	5.293 ± 0.151	4.498 ± 0.11	0.678 ± 0.011

Note: a, b, and c represent significant differences in the average clustering coefficient (avgCC), average path length (GD), and modularity (M) between empirical networks and random networks, respectively.

Table S2 Taxonomic classification of keystone ASVs.

Groups	ID	Network roles	Phylum	Class	Family
Day0	ASV100	Connector hubs	Proteobacteria	Gammaproteobacteria	Nitrosomonadaceae
	ASV169	Connector hubs	Planctomycetota	Planctomycetes	Isosphaeraceae
	ASV771	Connector hubs	Bdellovibrionota	Bdellovibrionia	Bacteriovoracaceae
C	ASV84	Module hubs	Proteobacteria	Gammaproteobacteria	Xanthomonadaceae
	ASV203	Module hubs	Proteobacteria	Gammaproteobacteria	Xanthomonadaceae
	ASV216	Module hubs	Acidobacteriota	Thermoanaerobaculia	Thermoanaerobaculaceae
	ASV338	Connector hubs	Proteobacteria	Gammaproteobacteria	Rhodocyclaceae
	ASV375	Connector hubs	unclassified	unclassified	unclassified
	ASV1324	Connector hubs	Myxococcota	Polyangia	Haliangiaceae
	ASV1332	Module hubs	Proteobacteria	Alphaproteobacteria	Acetobacteraceae
L	ASV34	Module hubs	Bacteroidota	Bacteroidia	Chitinophagaceae
	ASV79	Module hubs	Bdellovibrionota	Oligoflexia	unclassified
	ASV146	Connector hubs	Actinobacteriota	Acidimicrobiia	unclassified
	ASV180	Connector hubs	unclassified	unclassified	unclassified
	ASV202	Module hubs	Planctomycetota	Planctomycetes	Isosphaeraceae
	ASV724	Connector hubs	Proteobacteria	Alphaproteobacteria	Sphingomonadaceae
	ASV758	Connector hubs	Proteobacteria	Alphaproteobacteria	Sphingomonadaceae
	ASV1108	Module hubs	Chloroflexi	Dehalococcoidia	uncultured
	ASV1350	Connector hubs	Acidobacteriota	Acidobacteriae	unclassified
	ASV1547	Connector hubs	Planctomycetota	Planctomycetes	Gemmataceae
	ASV1585	Connector hubs	Chloroflexi	Ktedonobacteria	JG30_KF_AS9
	ASV2298	Connector hubs	Gemmatimonadota	Gemmatimonadetes	Gemmatimonadaceae
H	ASV12	Module hubs	Bacteroidota	Bacteroidia	env.OPS_17
	ASV41	Connector hubs	Gemmatimonadota	Gemmatimonadetes	Gemmatimonadaceae
	ASV224	Module hubs	Planctomycetota	Planctomycetes	Gemmataceae
	ASV801	Connector hubs	Planctomycetota	Planctomycetes	Isosphaeraceae
	ASV1013	Module hubs	Planctomycetota	Planctomycetes	Pirellulaceae
	ASV1523	Module hubs	Proteobacteria	Gammaproteobacteria	B1_7BS
	ASV1662	Connector hubs	Crenarchaeota	Nitrososphaeria	Nitrososphaeraceae
	ASV2259	Connector hubs	Planctomycetota	Planctomycetes	Isosphaeraceae

Table S3 Results of Monte Carlo permutation test.

**Properties**	**RDA1**	**RDA2**	**R^2**	**P-value**
E2	0.992	0.124	0.914	**0.010**
CH_4	-0.323	-0.946	0.912	**0.003**
